# Statistical Inference on the Shannon Entropy of Inverse Weibull Distribution under the Progressive First-Failure Censoring

**DOI:** 10.3390/e21121209

**Published:** 2019-12-10

**Authors:** Jiao Yu, Wenhao Gui, Yuqi Shan

**Affiliations:** School of Science, Beijing Jiaotong University, Beijing 100044, China; 17271108@bjtu.edu.cn (J.Y.); 17271089@bjtu.edu.cn (Y.S.)

**Keywords:** inverse Weibull distribution, entropy, progressive first-failure censored sample, maximum likelihood estimation, asymptotic interval, Lindley method, importance sampling procedure, highest posterior density credible interval

## Abstract

Entropy is an uncertainty measure of random variables which mathematically represents the prospective quantity of the information. In this paper, we mainly focus on the estimation for the parameters and entropy of an Inverse Weibull distribution under progressive first-failure censoring using classical (Maximum Likelihood) and Bayesian methods. For Bayesian approaches, the Bayesian estimates are obtained based on both asymmetric (General Entropy, Linex) and symmetric (Squared Error) loss functions. Due to the complex form of Bayes estimates, we cannot get an explicit solution. Therefore, the Lindley method as well as Importance Sampling procedure is applied. Furthermore, using Importance Sampling method, the Highest Posterior Density credible intervals of entropy are constructed. As a comparison, the asymptotic intervals of entropy are also gained. Finally, a simulation study is implemented and a real data set analysis is performed to apply the previous methods.

## 1. Introduction

Usually, in lifetime experiments, due to the restrictions of limited time and cost, accurate product lifetime data cannot be observed so we have censored data. The most common censoring schemes are so-called Type-I and Type-II censoring. In the first one, place *N* units in a life experiment and terminate the experiment after a predetermined time; for the other, terminate the experiment after the predetermined units number *m* has failed. Progressive censoring is a generalization of Type-II censoring which permits the units to be randomly removed at various time points instead of the end of the time.

Compared to conventional Type-I and Type-II censoring, progressive censoring, i.e., withdrawal of non-failed items, decreases the accuracy of estimation. However, in certain practical circumstances, experimenters are forced to withdraw items from tests. Thus, the application of the progressive censoring methodology allows profiting from information related to withdrawn items.

When the above methods still fail to meet the time and cost constraints, to further improve efficiency, other censoring schemes are successively filed by researchers. One of the successful attempts is the first failure censoring. In this censoring scheme, N=k×n units are assigned to *n* groups in random with *k* identical units in each group. The lifetime experiment is conducted by testing all groups simultaneously until the first failure is observed in each group.

Since progressive censoring and first-failure censoring can both greatly enhance the efficiency of the lifetime experiment, Ref. [1] united these two items and developed a novel censoring scheme called the progressive first-failure censoring. In this censoring, N=k×n samples are divided into *n* disjoint groups in random with *k* identical units at the beginning of the life experiment, and the experiment is terminated when the *m*th unit fails. When the *i*th unit fails, the group containing the *i*th is removed together with $R_{i}$ randomly selected groups, and when the *m*th fails, all the surviving groups are removed. Here, $R=(R_{1},\ldots ,R_{m})$ and *m* are set in advance. Note that

(1) When k=1, the progressive first failure censoring can be reduced to the well-known progressive Type-II censoring.

(2) When $R_{1}=R_{2}=\ldots =R_{m}=0$, this censoring becomes the mentioned first-failure censoring.

(3) When k=1, $R_{1}=R_{2}=\ldots =R_{m-1}=0$ and $R_{m}=n-m$, this censoring corresponds to Type-II censoring.

Since it is more efficient than other censoring schemes, many researchers have discussed the study of the progressive first-failure censoring. Ref. [2] considered both the point and interval estimation of two parameters from a Burr-XII distribution when both of the parameters are unknown; Ref. [3] dealt with the reliability function of GIED (Generalized inverted exponential distribution) under progressive first-failure censoring; Ref. [4] established different reliability sampling plans using two criteria from a Lognormal distribution based on the progressive first-failure censoring; Ref. [5] chose a competing risks data model under progressive first-failure censoring from a Gompertz distribution and estimated the model using Bayesian and non-Bayesian methods; Ref. [6] considered the lifetime performance index ($C_{L}$) under the progressive first-failure censoring schemes of a Pareto model, solved the problem of the hypothesis testing of $C_{L}$, and gave a lower specification limit.

The Weibull distribution is used in a widespread manner in analyzing lifetime data. Nevertheless, the Weibull distribution possesses a constant, decreasing or increasing failure rate function, its failure rate function cannot be non-monotone, such as unimodal. In practice, if the research shows that the empirical failure rate function is non-monotone, then the Inverse Weibull model is a more suitable choice than the Weibull model. The Inverse Weibull model has a wide variety of applications in pharmacy, economics and chemistry.

The cumulative distribution function and the probability density function of the Inverse Weibull distribution (IWD) are separately written as

(1) $$\begin{array}{c} F\left(x;\alpha ,\lambda \right)=e^{-\lambda x^{-\alpha }} \end{array}$$

and

(2) $$\begin{array}{c} f\left(x;\alpha ,\lambda \right)=\alpha \lambda e^{-\lambda x^{-\alpha }}x^{-\alpha -1}, \end{array}$$

where x>0,λ>0,α>0, λ is the scale parameter and α is the shape parameter.

The failure rate function is

$$\begin{array}{c} h\left(x;\alpha ,\lambda \right)=\frac{\alpha \lambda e^{-\lambda x^{-\alpha }}x^{-\alpha -1}}{1-e^{-\lambda x^{-\alpha }}}. \end{array}$$

**Figure 1:**
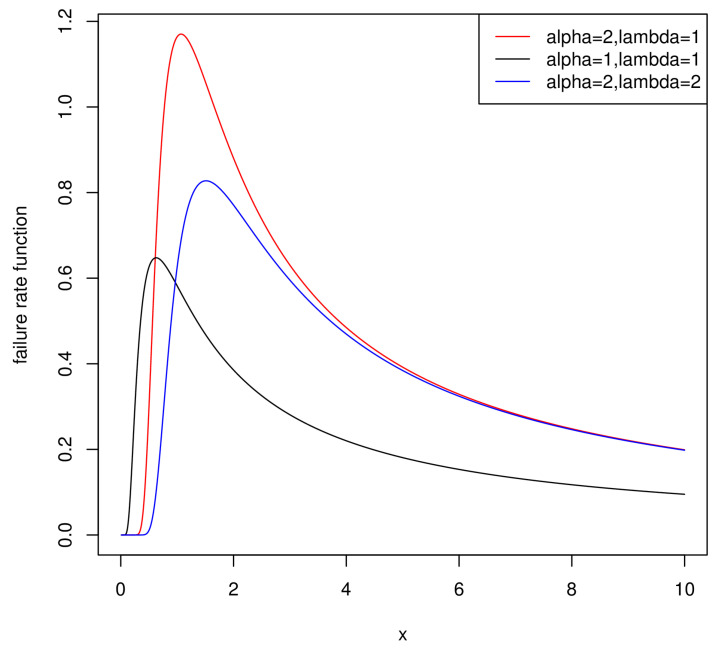
The failure rate function.

One of the most important properties of the IWD is that its failure rate function can be unimodal. Figure 1 also evidently supports this conclusion, and we can observe that the distribution whose failure rate function is unimodal is more flexible in application.

Many researchers have studied the Inverse Weibull distribution. Ref. [7] invesigated the Bayesian inference and successfully predicted the IWD for the type-II censoring scheme; Ref. [8] not only considered the Baysian estimation but also the generalized Bayesian estimation for the IWD parameters; Ref. [9] used three classical methods to estimate the parameters from IWD; Ref. [10] estimated the unknown parameters from IWD under the progressive type-I interval censoring and chose the optimal censoring schemes; Ref. [11] adopted two methods to get bias corrections of unknown parameters using maximum likelihood method of the IWD.

Entropy is a quantitive measure of the uncertainty of each probability distribution. For the random variable X, of the probability density distribution f(x), the Shannon entropy, recorded as H(X), is written as:

(3) $$\begin{array}{c} H\left(X\right)=-\int_{-\infty }^{\infty }f\left(x\right)\log\left(f\left(x\right)\right)\;dx. \end{array}$$

Many studies about entropy can be found in the literature. Ref. [12] proposed an indirect method using a decomposition to simplify the entropy’s calculation under the progressive Type-II censoring; Ref. [13] estimated the entropy for several exponential distributions and extended the results to other circumstances; Ref. [14] estimated the Shannon entropy of a Rayleigh model under doubly generalized Type-II hydrid censoring, and compared the performance by two criteria.

The Shannon entropy of the IWD is given by:

(4) $$\begin{array}{c} H\left(X\right)=\frac{\alpha +1}{\alpha }\left[\gamma +\log\left(\lambda \right)\right]+1-\log\left(\alpha \lambda \right), \end{array}$$

where γ is a Euler constant.

In this paper, we discuss the maximum likelihood and Bayesian estimation of the paramaters (α,λ) and entropy of IWD under progressive first-failure censoring. As far as we know, this topic is very new and few researchers study it. However, it needs in-depth research and innovation. The rest of this paper is elaborated as follows:

In Section 2, we derive the maximum likelihood estimation of entropy and parameters. In Section 3, we present the asymptotic intervals for the entropy and parameters. In Section 4, we work out the Bayesian estimation of entropy and parameters using Lindley and Importance Sampling methods. In Section 5, a simulation study is organized to compare different estimators. In Section 6, we analyze a real data set to explain the previous conclusions. Finally, in Section 7, a conclusion is presented.

## 2. Maximum Likelihood Estimation

We consider the maximum likelihood estimates (MLEs) for the entropy and parameters of an Inverse Weibull distribution under progressive first-failure censoring. Set $X_{1:m:n:k}^{R}\leq X_{2:m:n:k}^{R}\leq \ldots \leq X_{m:m:n:k}^{R}$ be a sample from IWD under the progressive first-failure censoring $\left(k,n,m,R_{1},\ldots ,R_{m}\right)$. For simplicity, we choose $x_{i}$ for representing $x_{i:m:n:k}^{R}$, i=1,…,m. The joint probability density function is

(5) $$\begin{array}{c} f_{X_{1:m:n:k}^{R}\ldots X_{m:m:n:k}^{R}}\left(x_{1}\ldots x_{m}\right)=Pk^{m}\prod_{i=1}^{m}f\left(x_{i}\right){\left[1-F\left(x_{i}\right)\right]}^{k(R_{i}+1)-1}, \end{array}$$

where $0<x_{1}<\ldots <x_{m}<\infty$ and $P=n\left(n-1-R_{1}\right)\ldots \left(n-m+1-R_{1}-\ldots -R_{m-1}\right)$ is a normalizing constant.

Combining (1), (2), and (5), the likelihood function (LF) is

(6) $$\begin{array}{c} L\left(x|\alpha ,\lambda \right)=Pk^{m}\alpha^{m}\lambda^{m}e^{-\lambda \sum_{i=1}^{m}x_{i}^{-\alpha }}\prod_{i=1}^{m}x_{i}^{-\alpha -1}\prod_{i=1}^{m}{\left(1-e^{-\lambda x_{i}^{-\alpha }}\right)}^{k(R_{i}+1)-1}. \end{array}$$

Then, the log-likelihood function is written as

(7) $$\begin{array}{ccc} l(x|\alpha ,\lambda ) & = & \log P+m\log k+m\log\alpha +m\log\lambda -\lambda \sum_{i=1}^{m}x_{i}^{-\alpha }-\left(\alpha +1\right)\sum_{i=1}^{m}\log x_{i}\\ & & +\sum_{i=1}^{m}\left(k\left(R_{i}+1\right)-1\right)\log\left(1-e^{-\lambda x_{i}^{-\alpha }}\right). \end{array}$$

For partial derivatives with respect to α and λ, the corresponding score equations are

(8) $$\begin{array}{c} \frac{\partial l}{\partial \alpha }=\frac{m}{\alpha }+\lambda \sum_{i=1}^{m}x_{i}^{-\alpha }\log x_{i}-\sum_{i=1}^{m}\log x_{i}-\sum_{i=1}^{m}\left(k\left(R_{i}+1\right)-1\right)\frac{\lambda x_{i}^{-\alpha }\log x_{i}e^{-\lambda x_{i}^{-\alpha }}}{1-e^{-\lambda x_{i}^{-\alpha }}}=0, \end{array}$$

(9) $$\begin{array}{c} \frac{\partial l}{\partial \lambda }=\frac{m}{\lambda }-\sum_{i=1}^{m}x_{i}^{-\alpha }+\sum_{i=1}^{m}\left(k\left(R_{i}+1\right)-1\right)\frac{x_{i}^{-\alpha }e^{-\lambda x_{i}^{-\alpha }}}{1-e^{-\lambda x_{i}^{-\alpha }}}=0. \end{array}$$

The MLEs $\hat{\alpha }$ and $\hat{\lambda }$, separately, are the roots of Equations (8) and (9). The equations don’t have an explicit solution, so we need some numerical techniques to approximate the values of these parameters. Furthermore, according to the invariance property of MLE, we derive the ML estimator of entropy as:

(10) $$\begin{array}{c} \hat{H}\left(X\right)=\frac{\hat{\alpha }+1}{\hat{\alpha }}\left[\gamma +\log\left(\hat{\lambda }\right)\right]+1-\log\left(\hat{\alpha }\hat{\lambda }\right). \end{array}$$

## 3. Confidence Intervals

### 3.1. Asymptotic Intervals for MLEs

The 100(1−ξ)% confidence intervals (CIs) for the two parameters α and λ can be constructed by the asymptotic normality of MLEs with $Var(\hat{\alpha })$ and $Var(\hat{\lambda })$ which are obtained by the inverse of the observed Fisher matrix.

From Equation (7), find second-order partial derivatives for α and λ as follows:

$$\begin{array}{ccc} l_{20}=\frac{\partial^{2}l}{\partial \alpha^{2}} & = & \sum_{i=1}^{m}\left(k\left(R_{i}+1\right)-1\right)\frac{\lambda x_{i}^{-\alpha }\log^{2}x_{i}e^{-\lambda x_{i}^{-\alpha }}\left(1-\lambda x_{i}^{-\alpha }-e^{-\lambda x_{i}^{-\alpha }}\right)}{{\left(1-e^{-\lambda x_{i}^{-\alpha }}\right)}^{2}}\\ & & -\frac{m}{\alpha^{2}}-\lambda \sum_{i=1}^{m}x_{i}^{-\alpha }{\left(\log x_{i}\right)}^{2},\\ l_{11}=\frac{\partial^{2}l}{\partial \alpha \partial \lambda } & = & \sum_{i=1}^{m}x_{i}^{-\alpha }\log x_{i}+\sum_{i=1}^{m}\left(k\left(R_{i}+1\right)-1\right)\frac{x_{i}^{-\alpha }\log x_{i}e^{-\lambda x_{i}^{-\alpha }}\left(-1+\lambda x_{i}^{-\alpha }+e^{-\lambda x_{i}^{-\alpha }}\right)}{{\left(1-e^{-\lambda x_{i}^{-\alpha }}\right)}^{2}},\\ l_{02}=\frac{\partial^{2}l}{\partial \lambda^{2}} & = & -\frac{m}{\lambda^{2}}+\sum_{i=1}^{m}\left(k\left(R_{i}+1\right)-1\right)\frac{-x_{i}^{-2\alpha }e^{-\lambda x_{i}^{-\alpha }}}{{\left(1-e^{-\lambda x_{i}^{-\alpha }}\right)}^{2}}. \end{array}$$

The Fisher information matrix of two parameters α and λ is I(α,λ). Here, we approximate that ${\left(\hat{\alpha },\hat{\lambda }\right)}^{T}$ is a bivariate normal vector with mean ${\left(\alpha ,\lambda \right)}^{T}$ and covariance matrix $I^{-1}=I^{-1}\left(\alpha ,\lambda \right)$. As a matter of fact, we use $I^{-1}\left(\hat{\alpha },\hat{\lambda }\right)$ to make an estimation of $I^{-1}\left(\alpha ,\lambda \right)$. In other words,

(11) $$\begin{array}{c} {\left(\hat{\alpha },\hat{\lambda }\right)}^{T}\overset{\mathrm{as}}{∼}N\left[{\left(\alpha ,\lambda \right)}^{T},I^{-1}\left(\hat{\alpha },\hat{\lambda }\right)\right], \end{array}$$

where

(12) $$\begin{array}{ccc} I^{-1}\left(\hat{\alpha },\hat{\lambda }\right) & = & {\left(\begin{array}{cc} -l_{20} & -l_{11}\\ -l_{11} & -l_{02} \end{array}\right)}_{\left(\alpha ,\lambda \right)=\left(\hat{\alpha },\hat{\lambda }\right)}^{-1}=\left(\begin{array}{cc} \tau_{11} & \tau_{12}\\ \tau_{21} & \tau_{22} \end{array}\right). \end{array}$$

Thus, based on the normal approximation, the 100(1−ξ)% CIs for two parameters α and λ are

(13) $$\begin{array}{c} \hat{\alpha }\pm Z_{\xi /2}\sqrt{\tau_{11}},\;\hat{\lambda }\pm Z_{\xi /2}\sqrt{\tau_{22}}. \end{array}$$

Here, $Z_{\xi /2}$ is the ξ/2 percentile of the standard normal distribution. Thus, as to obtain the approximate estimation of the variance of entropy, we use the delta method. Let

(14) $$\begin{array}{c} {\hat{\Psi }}^{\prime }=\left(\frac{\partial H}{\partial \alpha },\frac{\partial H}{\partial \lambda }\right), \end{array}$$

where

$$\begin{array}{ccc} \frac{\partial H}{\partial \alpha } & = & -\frac{\left(\alpha +1)(\gamma +\log(\lambda )\right)}{\alpha^{2}}+\frac{\gamma +\log(\lambda )}{\alpha }-\frac{1}{\alpha },\\ \frac{\partial H}{\partial \lambda } & = & \frac{\alpha +1}{\alpha \lambda }-\frac{1}{\lambda }. \end{array}$$

Then, the approximate estimate of $\hat{Var}\left(\hat{H}\right)$ is obtained by

(15) $$\begin{array}{c} \hat{Var}\left(\hat{H}\right)=\left[{\hat{\Psi }}^{\prime }I^{-1}\left(\hat{\alpha },\hat{\lambda }\right)\hat{\Psi }\right]. \end{array}$$

Therefore, we approximate that

(16) $$\begin{array}{c} \frac{\hat{H}-H}{\sqrt{\hat{Var}\left(\hat{H}\right)}}∼N\left(0,1\right). \end{array}$$

The asymptotic 100(1−ξ)% CI for entropy is derived as

(17) $$\begin{array}{c} \hat{H}\pm Z_{\xi /2}\sqrt{\hat{Var}\left(\hat{H}\right)}. \end{array}$$

### 3.2. Asymptotic Intervals for Log-Transformed MLE

Ref. [15] proposed that the asymptotic CI using log-transformed MLE has a more precise coverage probabilty. It is clear that α, λ, and entropy are all positive. Then, we obtain that 100(1−ξ)% asymptotic approximate CIs for log-transformed MLEs are

(18) $$\begin{array}{c} \log\left(\hat{\alpha }\right)\pm Z_{\xi /2}\sqrt{\tau_{11}\left(\log\left(\hat{\alpha }\right)\right)},\;\log\left(\hat{\lambda }\right)\pm Z_{\xi /2}\sqrt{\tau_{22}\left(\log\left(\hat{\lambda }\right)\right)}. \end{array}$$

Thus, based on the normal approximation of log-transformed MLE, the 100(1−ξ)% CIs for two parameters α and λ are

(19) $$\begin{array}{c} \hat{\alpha }\exp\left[\pm \frac{Z_{\xi /2}\sqrt{\tau_{11}}}{\hat{\alpha }}\right],\;\hat{\lambda }\exp\left[\pm \frac{Z_{\xi /2}\sqrt{\tau_{22}}}{\hat{\lambda }}\right]. \end{array}$$

Furthermore, a 100(1−ξ)% CI for entropy is

(20) $$\begin{array}{c} \hat{H}\exp\left[\pm \frac{Z_{\xi /2}\sqrt{\hat{Var}\left(\hat{H}\right)}}{\hat{H}}\right]. \end{array}$$

## 4. Bayes Estimation

### 4.1. Prior and Posterior Distribution

Both α and λ are unknown parameters, so we don’t have any conjugate prior for both α and λ. Usually, we choose independent priors of α and λ which are both Gamma distributions. However, for the Inverse Weibull distribution, it is not appropriate to choose gamma for both priors. The specific reason is explained in detail in the Importance Sampling procedure subsection. Thus, in this case, we consider the following prior distributions:

λ possesses a Gamma prior G(a,b) with the probability density function

(21) $$\begin{array}{c} \pi_{1}\left(\lambda \right)\propto \lambda^{a-1}e^{-b\lambda }, \end{array}$$

α has a non-informative prior with the following probability density function

(22) $$\begin{array}{c} \pi_{2}\left(\alpha \right)=\frac{1}{\alpha }, \end{array}$$

where *a* and *b* are pre-fixed to be known and positive.

Now, the joint prior distribution of the two parameters α and λ can be obtained by

(23) $$\begin{array}{c} \pi \left(\alpha ,\lambda \right)\propto \frac{\lambda^{a-1}e^{-b\lambda }}{\alpha }. \end{array}$$

Then, the joint posterior PDF of two parameters α and λ is derived by

(24) $$\begin{array}{c} \pi \left(\alpha ,\lambda |x\right)=\frac{L(x|\alpha ,\lambda )\pi (\alpha ,\lambda )}{\int_{0}^{\infty }\int_{0}^{\infty }L\left(x|\alpha ,\lambda \right)\pi \left(\alpha ,\lambda \right)\;d\alpha d\lambda }. \end{array}$$

### 4.2. Symmetric and Asymmetric Loss Functions

Choosing loss function is an important part of Bayesian inference. In this subsection, we consider the Bayes estimation for two parameters α, λ, and entropy of an IWD under both the asymmetric and symmetric loss functions. A widely used symmetric loss function is the squared error loss function (SELF). As for asymmetric loss functions, we choose the general entropy loss function (GELF) and linex loss function (LLF). The SELF, LLF, and GELF are defined as

$$\begin{array}{ccc} L_{S}\left(ℵ,\hat{ℵ}\right) & = & {\left(\hat{ℵ}-ℵ\right)}^{2},\\ L_{L}\left(ℵ,\hat{ℵ}\right) & = & \exp\left(p\left(\hat{ℵ}-ℵ\right)\right)-p\left(\hat{ℵ}-ℵ\right)-1,\\ L_{E}\left(ℵ,\hat{ℵ}\right) & = & {\left(\frac{\hat{ℵ}}{ℵ}\right)}^{-q}-q\log\left(\frac{\hat{ℵ}}{ℵ}\right)-1, \end{array}$$

where $\hat{ℵ}$ means an estimate of ℵ. In LLF and SELF, the symbols of *p* and *q* indicate the direction of the asymmetry, and their sizes mean the different level. Neither of them are zero.

The Bayes estimates of ℵ under above loss functions are

$$\begin{array}{ccc} {\hat{ℵ}}_{S} & = & E_{ℵ}\left(ℵ|x\right),\\ {\hat{ℵ}}_{L} & = & -\frac{1}{p}\log\left[E_{ℵ}\left(\exp\left(-pℵ\right)|x\right)\right],\\ {\hat{ℵ}}_{E} & = & {\left[E_{ℵ}\left({ℵ}^{-q}|x\right)\right]}^{-\frac{1}{q}}, \end{array}$$

where $E_{ℵ}$ means the posterior expectation under the parameter ℵ. Now, we can derive the Bayes estimates of α, λ, and entopy under SELF, LLF, and GELF.

To begin with, Bayes estimate of g(α,λ) under SELF is

(25) $$\begin{array}{c} \hat{g}{\left(\alpha ,\lambda \right)}_{S}=\frac{\int_{0}^{\infty }\int_{0}^{\infty }g\left(\alpha ,\lambda \right)L\left(x|\alpha ,\lambda \right)\pi \left(\alpha ,\lambda \right)\;d\alpha d\lambda }{\int_{0}^{\infty }\int_{0}^{\infty }L\left(x|\alpha ,\lambda \right)\pi \left(\alpha ,\lambda \right)\;d\alpha d\lambda }. \end{array}$$

Let g(α,λ) takes the value of α, λ, and entropy, then we can easily obtain the corresponding estimation under SELF.

Moreover, Bayes estimate of g(α,λ) under LLF is

(26) $$\begin{array}{c} \hat{g}{\left(\alpha ,\lambda \right)}_{L}=-\frac{1}{p}\log\left[\frac{\int_{0}^{\infty }\int_{0}^{\infty }e^{-pg(\alpha ,\lambda )}L\left(x|\alpha ,\lambda \right)\pi \left(\alpha ,\lambda \right)\;d\alpha d\lambda }{\int_{0}^{\infty }\int_{0}^{\infty }L\left(x|\alpha ,\lambda \right)\pi \left(\alpha ,\lambda \right)\;d\alpha d\lambda }\right]. \end{array}$$

Let g(α,λ) take the value of α, λ, and entropy; then, we can obviously obtain the corresponding estimation under LLF.

Finally, Bayes estimate of g(α,λ) under GELF is

(27) $$\begin{array}{c} \hat{g}{\left(\alpha ,\lambda \right)}_{E}={\left[\frac{\int_{0}^{\infty }\int_{0}^{\infty }g{\left(\alpha ,\lambda \right)}^{-q}L\left(x|\alpha ,\lambda \right)\pi \left(\alpha ,\lambda \right)\;d\alpha d\lambda }{\int_{0}^{\infty }\int_{0}^{\infty }L\left(x|\alpha ,\lambda \right)\pi \left(\alpha ,\lambda \right)\;d\alpha d\lambda }\right]}^{-\frac{1}{q}} \end{array}$$

Let g(α,λ) take the value of α, λ, and entropy; then, we can evidently obtain the corresponding esitimation under GELF.

Obviously, the Bayesian estimation cannot be accurately expressed in a closed form. Hence, we recommend using Lindley method as well as Importance Sampling procedure to derive the Bayesian estimation.

### 4.3. Lindley Approximation

The Bayes estimates are in the shape of a specific ratio of two integals which can’t be reduced to a closed form. Therefore, we utilize Lindley approximation method to derive the Bayes estimates. For the $\left({ℵ}_{1},{ℵ}_{2}\right)$, the Bayesian estimate is

(28) $$\begin{array}{c} \hat{g}=g\left({\hat{ℵ}}_{1},{\hat{ℵ}}_{2}\right)+0.5\left(S+l_{30}I_{12}+l_{03}I_{21}+l_{21}O_{12}+l_{12}O_{21}\right)+\rho_{1}S_{12}+\rho_{2}S_{21}, \end{array}$$

where

$$\begin{array}{c} S=\sum_{i=1}^{2}\sum_{j=1}^{2}\omega_{ij}\tau_{ij},\;l_{ij}=\frac{\partial_{i+j}l}{\partial {ℵ}_{1}^{i}\partial {ℵ}_{2}^{j}},\;i+j=3;\;i,j=0,1,2,3, \end{array}$$

$$\begin{array}{c} \rho_{i}=\frac{\partial \rho }{\partial {ℵ}_{i}},\;\omega_{i}=\frac{\partial g}{\partial {ℵ}_{i}},\;\omega_{ij}=\frac{\partial^{2}g}{\partial {ℵ}_{i}\partial {ℵ}_{j}},\;\rho =\log\pi \left({ℵ}_{1},{ℵ}_{2}\right), \end{array}$$

$$\begin{array}{c} S_{ij}=\omega_{i}\tau_{ii}+\omega_{j}\tau_{ji},\;I_{ij}=\omega_{ii}\left(\omega_{i}\tau_{ii}+\omega_{j}\tau_{ij}\right),\;O_{ij}=3\omega_{i}\tau_{ii}\tau_{ij}+\omega_{j}\left(\tau_{ii}\tau_{jj}+2{\tau_{ij}}^{2}\right). \end{array}$$

All terms are estimated by MLEs of ${ℵ}_{1}$ and ${ℵ}_{2}$. For our problem, we take $\hat{g}$ for the estimation of $\left({ℵ}_{1},{ℵ}_{2}\right)=\left(\alpha ,\lambda \right)$. Then, we obtain

$$\begin{array}{ccc} l_{30} & = & \frac{2m}{\alpha^{3}}+\lambda \sum_{i=1}^{m}x_{i}^{-\alpha }\log^{3}x_{i}+\sum_{i=1}^{m}\left(k\left(R_{i}+1\right)-1\right)(\frac{\lambda^{3}x_{i}^{-3\alpha }\log^{3}x_{i}e^{-\lambda x_{i}^{-\alpha }}}{1-e^{-\lambda x_{i}^{-\alpha }}}\\ & & -\frac{3\lambda^{3}x_{i}^{-3\alpha }\log^{3}x_{i}e^{-2\lambda x_{i}^{-\alpha }}}{{\left(1-e^{-\lambda x_{i}^{-\alpha }}\right)}^{2}}-\frac{2\lambda^{3}x_{i}^{-3\alpha }\log^{3}x_{i}e^{-3\lambda x_{i}^{-\alpha }}}{{\left(1-e^{-\lambda x_{i}^{-\alpha }}\right)}^{3}}),\\ l_{03} & = & \frac{2m}{\lambda^{3}}+\sum_{i=1}^{m}\left(k\left(R_{i}+1\right)-1\right)\frac{x_{i}^{-3\alpha }e^{-\lambda x_{i}^{-\alpha }}\left(1+e^{-\lambda x_{i}^{-\alpha }}\right)}{{\left(1-e^{-\lambda x_{i}^{-\alpha }}\right)}^{3}},\\ l_{21} & = & -\sum_{i=1}^{m}x_{i}^{-\alpha }\log^{2}x_{i}+\sum_{i=1}^{m}\left(k\left(R_{i}+1\right)-1\right)\left(\frac{\lambda^{2}x_{i}^{-3\alpha }\log^{2}x_{i}e^{-\lambda x_{i}^{-\alpha }}}{1-e^{-\lambda x_{i}^{-\alpha }}}\right.\\ & & \left.+\frac{3\lambda^{2}x_{i}^{-3\alpha }\log^{2}x_{i}e^{-2\lambda x_{i}^{-\alpha }}}{{\left(1-e^{-\lambda x_{i}^{-\alpha }}\right)}^{2}}+\frac{2\lambda^{2}x_{i}^{-3\alpha }\log^{2}x_{i}e^{-3\lambda x^{-\alpha }}}{{\left(1-e^{-\lambda x_{i}^{-\alpha }}\right)}^{3}}-\frac{3\lambda x_{i}^{-2\alpha }\log^{2}x_{i}e^{-\lambda x_{i}^{-\alpha }}}{1-e^{-\lambda x_{i}^{-\alpha }}}\right.\\ & & \left.-\frac{3\lambda x_{i}^{-2\alpha }\log^{2}x_{i}e^{-2\lambda x_{i}^{-\alpha }}}{{\left(1-e^{-\lambda x_{i}^{-\alpha }}\right)}^{2}}+\frac{x_{i}^{-\alpha }\log^{2}x_{i}e^{-\lambda x_{i}^{-\alpha }}}{1-e^{-\lambda x_{i}^{-\alpha }}}\right), \end{array}$$

$$\begin{array}{ccc} l_{12} & = & \sum_{i=1}^{m}\left(k\left(R_{i}+1\right)-1\right)\frac{x_{i}^{-3\alpha }\log x_{i}e^{-\lambda x_{i}^{-\alpha }}\left(2x_{i}^{\alpha }\left(e^{-\lambda x_{i}^{-\alpha }}-1\right)-\lambda \left(e^{-\lambda x_{i}^{-\alpha }}+1\right)\right)}{{\left(e^{-\lambda x_{i}^{-\alpha }}-1\right)}^{3}},\\ \rho_{1} & = & -\frac{1}{{\hat{\alpha }}^{2}},\;\rho_{2}=\frac{a-1}{\hat{\lambda }}-b. \end{array}$$

(1) Squared error loss function

Taking g(α,λ)=α or λ, the Bayesian estimates of two parameters α and λ under SELF, separately, are obtained by

(29) $$\begin{array}{ccc} {\hat{\alpha }}_{S} & = & \hat{\alpha }+0.5\left[\tau_{11}^{2}l_{30}+\tau_{21}\tau_{22}l_{03}+3\tau_{11}\tau_{12}l_{21}+\left(\tau_{22}\tau_{11}+2\tau_{21}^{2}\right)l_{12}\right]+\tau_{11}\rho_{1}+\tau_{12}\rho_{2}, \end{array}$$

(30) $$\begin{array}{ccc} {\hat{\lambda }}_{S} & = & \hat{\lambda }+0.5\left[\tau_{11}\tau_{12}l_{30}+\tau_{22}^{2}l_{03}+\left(\tau_{11}\tau_{22}+2\tau_{12}^{2}\right)l_{21}+3\tau_{22}\tau_{21}l_{12}\right]+\tau_{21}\rho_{1}+\tau_{22}\rho_{2}. \end{array}$$

Next, we derive Bayes estimate of entropy under SELF. We consider that

$$\begin{array}{ccc} g(\alpha ,\lambda ) & = & \frac{\alpha +1}{\alpha }\left[\gamma +\log\left(\lambda \right)\right]+1-\log\left(\alpha \lambda \right),\\ \omega_{1} & = & -\frac{\left(\alpha +1)(\gamma +\log(\lambda )\right)}{\alpha^{2}}+\frac{\gamma +\log(\lambda )}{\alpha }-\frac{1}{\alpha },\\ \omega_{2} & = & \frac{\alpha +1}{\alpha \lambda }-\frac{1}{\lambda },\\ \omega_{11} & = & \frac{1}{\alpha^{2}}+\left(\frac{2(\alpha +1)}{\alpha^{3}}-\frac{2}{\alpha^{2}}\right)\left(\gamma +\log\left(\lambda \right)\right),\\ \omega_{22} & = & \frac{1}{\lambda^{2}}-\frac{\alpha +1}{\alpha \lambda^{2}},\\ \omega_{12} & = & \omega_{21}=\frac{1}{\alpha \lambda }-\frac{\alpha +1}{\alpha^{2}\lambda }. \end{array}$$

The Bayes estimator of entropy can be obtained as earlier, and it is given by

(31) $$\begin{array}{ccc} {\hat{H}}_{S}\left(X\right) & = & \hat{H}\left(X\right)+0.5[\omega_{11}\tau_{11}+2\omega_{12}\tau_{12}+\omega_{22}\tau_{22}+l_{30}\left(\omega_{1}\tau_{11}^{2}+\omega_{2}\tau_{11}\tau_{12}\right)\\ & & +l_{21}\left(3\omega_{1}\tau_{11}\tau_{12}+\omega_{2}\left(\tau_{11}\tau_{22}+2\tau_{12}^{2}\right)\right)+l_{12}\left(3\omega_{2}\tau_{22}\tau_{21}+\omega_{1}\left(\tau_{22}\tau_{11}+2\tau_{21}^{2}\right)\right)\\ & & +l_{03}\left(\omega_{2}\tau_{22}^{2}+\omega_{1}\tau_{21}\tau_{22}\right)]+\rho_{1}\left(\omega_{1}\tau_{11}+\omega_{2}\tau_{21}\right)+\rho_{2}\left(\omega_{2}\tau_{22}+\omega_{1}\tau_{12}\right). \end{array}$$

(2) Linex loss function

For parameter α, taking g(α,λ)=α, we can obtain that

$$\begin{array}{c} \omega_{1}=-pe^{-p\alpha },\;\omega_{11}=p^{2}e^{-p\alpha },\;\omega_{12}=\omega_{21}=\omega_{22}=\omega_{2}=0. \end{array}$$

Utilizing the above expression in Equation (28), the Bayesian estimate of α is derived by

(32) $$\begin{array}{ccc} {\hat{\alpha }}_{L} & = & -\frac{1}{p}\log\{e^{-p\hat{\alpha }}+0.5[\omega_{11}\tau_{11}+\omega_{1}\tau_{11}^{2}l_{30}+\omega_{1}\tau_{21}\tau_{22}l_{03}+3\omega_{1}\tau_{11}\tau_{12}l_{21}\\ & & +\left(\tau_{22}\tau_{11}+2\tau_{21}^{2}\right)\omega_{1}l_{12}]+\omega_{1}\tau_{11}\rho_{1}+\omega_{1}\tau_{12}\rho_{2}\}. \end{array}$$

The Bayesian estimate of λ is obtained likewise:

(33) $$\begin{array}{ccc} {\hat{\lambda }}_{L} & = & -\frac{1}{p}\log\{e^{-p\hat{\lambda }}+0.5[\omega_{22}\tau_{22}+\omega_{2}\tau_{11}\tau_{12}l_{30}+\omega_{2}\tau_{22}^{2}l_{03}+\left(\tau_{11}\tau_{22}+2\tau_{12}^{2}\right)\omega_{2}l_{21}\\ & & +3\tau_{22}\tau_{21}\omega_{2}l_{12}]+\omega_{2}\tau_{21}\rho_{1}+\omega_{2}\tau_{22}\rho_{2}\}. \end{array}$$

Next, we derive the Bayesian estimate of entropy. It is clear that

$$\begin{array}{ccc} g(\alpha ,\lambda ) & = & e^{-pH(X)},\\ \omega_{1} & = & -pe^{-pH}\left[-\frac{\left(\alpha +1)(\gamma +\log(\lambda )\right)}{\alpha^{2}}+\frac{\gamma +\log(\lambda )}{\alpha }-\frac{1}{\alpha }\right],\\ \omega_{2} & = & -pe^{-pH}\left(\frac{\alpha +1}{\alpha \lambda }-\frac{1}{\lambda }\right),\\ \omega_{11} & = & p^{2}e^{-pH}{\left[-\frac{\left(\alpha +1)(\gamma +\log(\lambda )\right)}{\alpha^{2}}+\frac{\gamma +\log(\lambda )}{\alpha }-\frac{1}{\alpha }\right]}^{2}\\ & & -pe^{-pH}\left[\frac{2(\alpha +1)(\gamma +\log(\lambda ))}{\alpha^{3}}-\frac{2(\gamma +\log(\lambda ))}{\alpha^{2}}+\frac{1}{\alpha^{2}}\right],\\ \omega_{22} & = & p^{2}e^{-pH}{\left(\frac{\alpha +1}{\alpha \lambda }-\frac{1}{\lambda }\right)}^{2}-pe^{-pH}\left(-\frac{\alpha +1}{\alpha \lambda^{2}}+\frac{1}{\lambda^{2}}\right),\\ \omega_{12} & = & \omega_{21}=p^{2}e^{-pH}\left(\frac{\alpha +1}{\alpha \lambda }-\frac{1}{\lambda }\right)\left[-\frac{\left(\alpha +1)(\gamma +\log(\lambda )\right)}{\alpha^{2}}+\frac{\gamma +\log(\lambda )}{\alpha }-\frac{1}{\alpha }\right]\\ & & -pe^{-pH}\left(\frac{1}{\alpha \lambda }-\frac{\alpha +1}{\alpha^{2}\lambda }\right). \end{array}$$

The requested estimation of entropy can be derived in a similar method.

(34) $$\begin{array}{ccc} {\hat{H}}_{L}\left(X\right) & = & -\frac{1}{p}\log\{e^{-p\hat{H}}+0.5[\omega_{11}\tau_{11}+2\omega_{12}\tau_{12}+\omega_{22}\tau_{22}+l_{30}\left(\omega_{1}\tau_{11}^{2}+\omega_{2}\tau_{11}\tau_{12}\right)\\ & & +l_{12}\left(3\omega_{2}\tau_{22}\tau_{21}+\omega_{1}\left(\tau_{22}\tau_{11}+2\tau_{21}^{2}\right)\right)+l_{21}\left(3\omega_{1}\tau_{11}\tau_{12}+\omega_{2}\left(\tau_{11}\tau_{22}+2\tau_{12}^{2}\right)\right)\\ & & l_{03}\left(\omega_{2}\tau_{22}^{2}+\omega_{1}\tau_{21}\tau_{22}\right)]+\rho_{1}\left(\omega_{1}\tau_{11}+\omega_{2}\tau_{21}\right)+\rho_{2}\left(\omega_{2}\tau_{22}+\omega_{1}\tau_{12}\right)\}. \end{array}$$

(3) General entropy loss function

For the parameter α,

$$\begin{array}{ccc} g(\alpha ,\lambda ) & = & \alpha^{-q},\;\omega_{1}=-q\alpha^{-q-1},\;\omega_{11}=q\left(q+1\right)\alpha^{-q-2},\;\omega_{12}=\omega_{21}=\omega_{22}=\omega_{2}=0. \end{array}$$

Applying the above expression in Equation (28), the Bayesian estimate of α is derived by

(35) $$\begin{array}{ccc} {\hat{\alpha }}_{E} & = & \{{\hat{\alpha }}^{-q}+0.5\left[\omega_{11}\tau_{11}+\omega_{1}\tau_{11}^{2}l_{30}+\omega_{1}\tau_{21}\tau_{22}l_{03}+3\omega_{1}\tau_{11}\tau_{12}l_{21}+\left(\tau_{22}\tau_{11}+2\tau_{21}^{2}\right)\omega_{1}l_{12}\right].\\ & & +\omega_{1}\tau_{11}\rho_{1}+\omega_{1}\tau_{12}\rho_{2}{\}}^{-\frac{1}{q}}. \end{array}$$

The approximate Bayes estimator of λ is computed likewise.

(36) $$\begin{array}{ccc} {\hat{\lambda }}_{E} & = & \{{\hat{\lambda }}^{-q}+0.5\left[\omega_{22}\tau_{22}+\omega_{2}\tau_{11}\tau_{12}l_{30}+\omega_{2}\tau_{22}^{2}l_{03}+\left(\tau_{11}\tau_{22}+2\tau_{12}^{2}\right)\omega_{2}l_{21}+3\tau_{22}\tau_{21}\omega_{2}l_{12}\right].\\ & & +\omega_{2}\tau_{21}\rho_{1}+\omega_{2}\tau_{22}\rho_{2}{\}}^{-\frac{1}{q}}. \end{array}$$

Finally, we derive Bayesian estimate of entropy under GELF:

$$\begin{array}{ccc} g(\alpha ,\lambda ) & = & H{\left(X\right)}^{-q},\\ \omega_{1} & = & -qH^{-q-1}\left[-\frac{\left(\alpha +1)(\gamma +\log(\lambda )\right)}{\alpha^{2}}+\frac{\gamma +\log(\lambda )}{\alpha }-\frac{1}{\alpha }\right],\\ \omega_{2} & = & -qH^{-q-1}\left(\frac{\alpha +1}{\alpha \lambda }-\frac{1}{\lambda }\right),\\ \omega_{11} & = & q\left(q+1\right)H^{-q-2}{\left[-\frac{\left(\alpha +1)(\gamma +\log(\lambda )\right)}{\alpha^{2}}+\frac{\gamma +\log(\lambda )}{\alpha }-\frac{1}{\alpha }\right]}^{2}\\ & & -qH^{-q-1}\left[\frac{2(\alpha +1)(\gamma +\log(\lambda ))}{\alpha^{3}}-\frac{2(\gamma +\log(\lambda ))}{\alpha^{2}}+\frac{1}{\alpha^{2}}\right],\\ \omega_{22} & = & q\left(q+1\right)H^{-q-2}{\left(\frac{\alpha +1}{\alpha \lambda }-\frac{1}{\lambda }\right)}^{2}-qH^{-q-1}\left(-\frac{\alpha +1}{\alpha \lambda^{2}}+\frac{1}{\lambda^{2}}\right),\\ \omega_{12} & = & \omega_{21}=q\left(q+1\right)H^{-q-2}\left(\frac{\alpha +1}{\alpha \lambda }-\frac{1}{\lambda }\right)\left[-\frac{\left(\alpha +1)(\gamma +\log(\lambda )\right)}{\alpha^{2}}+\frac{\gamma +\log(\lambda )}{\alpha }-\frac{1}{\alpha }\right]\\ & & -qH^{-q-1}\left(\frac{1}{\alpha \lambda }-\frac{\alpha +1}{\alpha^{2}\lambda }\right). \end{array}$$

The Bayes estimator of entropy under GELF can be calculated as earlier, and it is given by

(37) $$\begin{array}{ccc} {\hat{H}}_{E}\left(X\right) & = & \{\hat{H}{\left(X\right)}^{-q}+0.5[\omega_{11}\tau_{11}+2\omega_{12}\tau_{12}+\omega_{22}\tau_{22}+l_{30}\left(\omega_{1}\tau_{11}^{2}+\omega_{2}\tau_{11}\tau_{12}\right)\\ & & +l_{21}\left(3\omega_{1}\tau_{11}\tau_{12}+\omega_{2}\left(\tau_{11}\tau_{22}+2\tau_{12}^{2}\right)\right)+l_{12}\left(3\omega_{2}\tau_{22}\tau_{21}+\omega_{1}\left(\tau_{22}\tau_{11}+2\tau_{21}^{2}\right)\right)\\ & & l_{03}\left(\omega_{2}\tau_{22}^{2}+\omega_{1}\tau_{21}\tau_{22}\right){]+\rho_{1}\left(\omega_{1}\tau_{11}+\omega_{2}\tau_{21}\right)+\rho_{2}\left(\omega_{2}\tau_{22}+\omega_{1}\tau_{12}\right)\}}^{-\frac{1}{q}}. \end{array}$$

### 4.4. Importance Sampling Procedure

Using the Lindley approximation method, we can get the Bayesian estimates of the unknown parameters and entropy. Although the Lindley method can make point estimation, it cannot determine the Highest Posterior Density (HPD) credible intervals. Thus, we recommend using the Importance Sampling to get Bayesian estimates and to derive HPD credible intervals as well.

To begin with, let’s solve the doubts before. If we choose two Gammas for prior distributions, record it as α∼G(a,b) and λ∼G(c,d). Then, the joint prior distribution is

$$\begin{array}{c} \pi \left(\alpha ,\lambda \right)\propto \alpha^{-a-1}e^{-b\alpha }\lambda^{-c-1}e^{-d\lambda }. \end{array}$$

Correspondingly, the joint posterior distribution is

$$\begin{array}{ccc} \pi (\alpha ,\lambda |X) & \propto & \alpha^{m+a-1}\lambda^{m+c-1}e^{-b\alpha -d\lambda }e^{-\lambda \sum_{i=1}^{m}x_{i}^{-\alpha }}\prod_{i=1}^{m}x_{i}^{-\alpha }\prod_{i=1}^{m}{\left(1-e^{-\lambda x_{i}^{-\alpha }}\right)}^{k(R_{i}+1)-1}\\ & = & \alpha^{(m+a)-1}e^{-\alpha (b+\sum_{i=1}^{m}\log\left(x_{i}\right))}\lambda^{(m+c)-1}e^{-\lambda (d+\sum_{i=1}^{m}x_{i}^{-\alpha })}\prod_{i=1}^{m}{\left(1-e^{-\lambda x_{i}^{-\alpha }}\right)}^{k(R_{i}+1)-1}\\ & = & G_{\alpha }\left(m+a,b+\sum_{i=1}^{m}\log\left(x_{i}\right)\right)G_{\lambda |\alpha }\left(m+c,d+\sum_{i=1}^{m}x_{i}^{-\alpha }\right)Q\left(\alpha ,\lambda \right), \end{array}$$

where $Q\left(\alpha ,\lambda \right)=\frac{\prod_{i=1}^{m}{\left(1-e^{-\lambda x_{i}^{-\alpha }}\right)}^{k(R_{i}+1)-1}}{{\left(d+\sum_{i=1}^{m}x_{i}^{-\alpha }\right)}^{m+c}}$.

We observe that $G_{\alpha }$ seems like Gamma distribution, but the second parameter $b+\sum_{i=1}^{m}\log\left(x_{i}\right)$ can not be proven to be strictly positive, so it cannot be considered to be a Gamma distribution. Obviously, it is not possible to generate its random samples according to the Gamma distribution, and it is also difficult to generate its random samples using other methods. Therefore, it is not appropriate to choose both Gammas as priors.

Then, we return to the prior distribution we selected before. To implement the Importance Sampling, the joint posterior distribution can be adapted as

$$\begin{array}{ccc} \pi (\alpha ,\lambda |X) & \propto & \alpha^{m-1}\lambda^{m+a-1}e^{-b\lambda }e^{-\lambda \sum_{i=1}^{m}x_{i}^{-\alpha }}\prod_{i=1}^{m}x_{i}^{-\alpha }\prod_{i=1}^{m}{\left(1-e^{-\lambda x_{i}^{-\alpha }}\right)}^{k(R_{i}+1)-1}\\ & = & \frac{{\left(b+\sum_{i=1}^{m}x_{i}^{-\alpha }\right)}^{m+a}}{\Gamma (m+a)}\lambda^{m+a-1}e^{-\lambda (b+\sum_{i=1}^{m}x_{i}^{-\alpha })}\times \frac{\alpha^{m-1}\prod_{i=1}^{m}x_{i}^{-\alpha }}{{\left(b+\sum_{i=1}^{m}x_{i}^{-\alpha }\right)}^{m+a}}\\ & & \times \prod_{i=1}^{m}{\left(1-e^{-\lambda x_{i}^{-\alpha }}\right)}^{k(R_{i}+1)-1}\\ & = & f_{1}\left(\lambda |\alpha \right)f_{2}\left(\alpha \right)Q\left(\alpha ,\lambda \right), \end{array}$$

where $f_{1}\left(\alpha ,\lambda \right)$ is a Gamma function $G(m+a,b+\sum_{i=1}^{m}x_{i}^{-\alpha })$ and

(38) $$\begin{array}{c} f_{2}\left(\alpha \right)=K\frac{\alpha^{m-1}\prod_{i=1}^{m}x_{i}^{-\alpha }}{{\left(b+\sum_{i=1}^{m}x_{i}^{-\alpha }\right)}^{m+a}}. \end{array}$$

Here, K is a normalizing constant and

(39) $$\begin{array}{c} Q\left(\alpha ,\lambda \right)=\prod_{i=1}^{m}{\left(1-e^{-\lambda x_{i}^{-\alpha }}\right)}^{k(R_{i}+1)-1}. \end{array}$$

Note that, in order to get the Bayesian estimates of parameters using the Importance Sampling, we demand to produce corresponding samples from $f_{1}\left(\lambda |\alpha \right)$ and $f_{2}\left(\alpha \right)$. It is uncomplicated and clear to produce samples from $f_{1}\left(\lambda |\alpha \right)$ because it is a simple Gamma distribution. As for producing samples from $f_{2}\left(\alpha \right)$, we have a Lemma.

**Lemma** **1.** *$f_{2}\left(\alpha \right)$ is log-concave.*

**Proof.**  $$\begin{array}{ccc} \log(f_{2}\left(\alpha \right)) & \propto & \left(m-1\right)\log\left(\alpha \right)-\alpha \sum_{i=1}^{m}\log\left(x_{i}\right)-\left(m+a\right)\log\left(b+\sum_{i=1}^{m}x_{i}^{-\alpha }\right)\\ \frac{\partial^{2}\log\left(f_{2}\left(\alpha \right)\right)}{\partial \alpha^{2}} & = & -\frac{m-1}{\alpha^{2}}-\left(m+a\right)\sum_{i=1}^{m}\frac{x_{i}^{-\alpha }\log^{2}\left(x_{i}\right)}{{\left(b+\sum_{i=1}^{m}x_{i}^{-\alpha }\right)}^{2}}. \end{array}$$

Since m is a postive number, the second-order partial derivative of $\log(f_{2}\left(\alpha \right))$ is constantly negative. Thereby, $f_{2}\left(\alpha \right)$ is log-concave. □

Then, using the approach originally proposed by [16], we can easily produce samples from $f_{2}\left(\alpha \right)$.

Using the following steps, we can produce several samples from the request scenario:

1. Produce α from $f_{2}\left(\alpha \right)$.
2. Produce λ from $G_{\lambda |\alpha }\left(m+a,b+\sum_{i=1}^{m}x_{i}^{-\alpha }\right)$.
3. Repeat Step 1 and 2 to derive $\left(\alpha_{1},\lambda_{1}\right),\left(\alpha_{2},\lambda_{2}\right),\ldots ,\left(\alpha_{M},\lambda_{M}\right)$.

Then, the required Bayesian estimate of ℧(α,λ) can be represented by

(40) $$\begin{array}{c} \frac{\sum_{i=1}^{M}℧\left(\alpha_{i},\lambda_{i}\right)Q\left(\alpha_{i},\lambda_{i}\right)}{\sum_{i=1}^{M}Q\left(\alpha_{i},\lambda_{i}\right)}. \end{array}$$

Furthermore, samples produced above can also be chosen to establish the HPD intervals for the parameters and entropy. Suppose that 0<p<1, and ${℧}_{p}$ makes $P(℧\left(\alpha ,\lambda \right)\leq {℧}_{p})=p$. For a given *p*, we purpose an approach to make a estimation of ${℧}_{p}$ and then to establish the HPD intervals for ℧(α,λ).

Firstly, we suppose

(41) $$\begin{array}{c} \vartheta_{i}=\frac{Q(\alpha_{i},\lambda_{i})}{\sum_{i=1}^{M}Q\left(\alpha_{i},\lambda_{i}\right)},\;i=1,\ldots ,M. \end{array}$$

For simplicity, we replace $℧(\alpha_{i},\lambda_{i})$ with ${℧}_{i}$. Then, sort $\left\{\left({℧}_{1},\vartheta_{1}\right),\ldots ,\left({℧}_{M},\vartheta_{M}\right)\right\}$ into $\left\{\left({℧}_{\left(1\right)},\vartheta_{\left(1\right)}\right),\ldots ,\left({℧}_{\left(M\right)},\vartheta_{\left(M\right)}\right)\right\}$, where ${℧}_{\left(1\right)}<\ldots <\vartheta_{\left(M\right)}$ and ${℧}_{\left(i\right)}$ is related to $\vartheta_{\left(i\right)}$ for i=1,…,M. Then, the Bayesian estimate of ${℧}_{p}$ is ${\hat{℧}}_{p}={℧}_{\left(M_{p}\right)}$, where $M_{p}$ is an integer which satifies

(42) $$\begin{array}{c} \sum_{i=1}^{M_{p}}\vartheta_{\left(i\right)}\leq p\leq \sum_{i=1}^{M_{p}+1}\vartheta_{\left(i\right)}. \end{array}$$

Therefore, a 100(1−ξ)% HPD interval of ℧(α,λ) can be derived by $\left({\hat{℧}}_{\delta },{\hat{℧}}_{\delta +1-\xi }\right)$ for $\delta =\vartheta_{\left(1\right)},\vartheta_{\left(1\right)}+\vartheta_{\left(2\right)},\ldots ,\sum_{i=1}^{M_{1-\xi }}\vartheta_{\left(i\right)}$. Finally, a 100(1−ξ)% HPD interval of ℧(α,λ) transforms $\left({\hat{℧}}_{\delta^{*}},{\hat{℧}}_{\delta^{*}+1-\xi }\right)$, where $\delta^{*}$ makes

(43) $$\begin{array}{c} {\hat{℧}}_{\delta^{*}+1-\beta }\leq {\hat{℧}}_{\delta +1-\beta }-{\hat{℧}}_{\delta } \end{array}$$

for all δ.

The next section will use Monte Carlo simulation to numerically and systematically compare previously proposed estimators.

## 5. Simulation Results

We will use the Monte Carlo simulation method to analyze the behavior of different estimators obtained by the above sections based on the expected value (EV) and mean squared error (MSE). The progressive first-failure censored samples are produced from different censoring schemes of $\left(k,n,m,R_{1},\ldots ,R_{m}\right)$ and various parameter values from the IWD by using the algorithm originally proposed by [17].

In general, we let α=2, λ=1, and correspondingly the entropy is 1.172676. We use the ‘optim’ command in the R software (version 3.6.1, Lucent Technologies, Mary Hill, NJ, USA) to get the approximate MLEs of α, λ, and entropy presented in Table 1. The Bayesian estimates under both asymmetric and symmetric loss functions are precisely computed by the Lindley method and Importance Samplings. For the Bayes estimation, we assign the value of hyperparameters as a=1,b=1 for Table 2, Table 3, Table 4, Table 5, Table 6 and Table 7 and a=0,b=0 for Table 8 and Table 9. Under the LLF, we let p=0.5 and p=1. Under the GELF, we choose q=−0.1 and q=1. We derive 95% asymmetric intervals of parameters using the MLEs and log-transformed MLEs and 95% HPD intervals. Pay attention that, for simplicity, the censoring schemes are presented by abbreviations such as (0∗5) represents (0,0,0,0,0) and ((1,0)∗2) represents (1,0,1,0). Table 2, Table 3, Table 4, Table 5 and Table 6, Table 8 present the Bayes estimation of α, λ, and entropy using the Lindley method. The Bayes estimation based on Importance Samplings is shown in Table 7 and Table 9. In Table 10, the interval estimation of entropy is presented.

As a whole, the EVs and MSEs of parameters and entropy all significantly decrease as the sample size *n* increases. In Table 1, Table 2, Table 3, Table 4, Table 5, Table 6, Table 7, Table 8 and Table 9, set *m* and *n* invariant, the EVs and MSEs of parameters and entropy both decrease as the group size *k* increases. Furthermore, set *k* and *n* invariant, the EVs and MSEs of parameters and entropy both decrease as *m* increases. Bayesian estimates with a=1,b=1 perform more precise than a=0,b=0, which is so-called non-informative. Using MLE and Bayes estimation based on the Lindley method is better than the Importance Sampling procedure. Bayes estimation using the Lindley method is a little bit more precise than the MLE. For LLF, choosing p=1 seems to be better than p=0.5. For GELF, q=−1 competes as well as q=1. In Table 7 and Table 9, we observe that the few censoring schemes such as (0∗24,25) and (0∗34,35) do not compete well.

In Table 10, the average length (AL) narrows down as the sample size *n* increases. Moreover, HPD intervals are more precise than confidence intervals based on AL. For confidence intervals, using log-transformed MLEs performs much better than MLEs. In almost all circumstances, the coverage probability (CP) of entropy derived here achieve their specified confidence intervals.

## 6. Real Data Analysis

We will analyze a real data set and apply the approaches put forward in the sections above. The data set was analyzed by [7,18]. The data show the surviving days of guinea pig injected with vairous species of tubercle bacilli. The quantity of regimen is the logarithmic of the quantity of bacillary units in 0.5 mL of the challenging solution. The sample size is 72 which are listed below: 12, 15, 22, 24, 24, 32, 32, 33, 34, 38, 38, 43, 44, 48, 52, 53, 54, 54, 55, 56, 57, 58, 58, 59, 60, 60, 60, 60, 61, 62, 63, 65, 65, 67, 68, 70, 70, 72, 73, 75, 76, 76, 81, 83, 84, 85, 87, 91, 95, 96, 98, 99, 109, 110, 121, 127, 129, 131, 143, 146, 146, 175, 175, 211, 233, 258, 258, 263, 297, 341, 341, 376 (unit: days).

Before analyzing the data, we want to test if the IWD matches the complete data well. To begin with, from [7], we conclude that the failure rate function of this data are unimodal, so it is scientific and reasonable to analyze the data using IWD. Then, we choose various approaches to analyze the goodness of fit of IWD using the MLE. We compute the −ln(L) and Kolmogorov–Smirnov (K–S) statistics with its associated *p*-value represented in Table 11. According to the *p*-value, the IWD fits the complete data well.

Now, we can consider the censoring data to illustrate the previous approaches. To generate the first-failure censored sample, we randomly sort the given data into n=36 groups with k=2 identical units in each group, and we can get the first-failure censored sample: 12, 15, 22, 24, 32, 32, 33, 34, 38, 38, 43, 44, 48, 52, 54, 55, 56, 58, 58, 60, 60, 61, 63, 65, 65, 68, 70, 70, 73, 76, 84, 91, 109, 110, 129, 143. Then, we produce samples using three diffrent progressive first-failure censoring which are (18,0∗17), (1∗18) and (0∗17,18) from the above sample with m=18. The results are organized in Table 12.

In Table 13, for MLE, we calculate the EVs, MSEs, and confidence intervals of the parameters and entropy; for Bayes estimation, we obtain the EVs, MSEs, and HPD intervals of entropy and two parameters. The estimates of α, λ, and entropy using the MLE and the Importance Sampling method are relatively close.

## 7. Conclusions

In this article, the problem of statistical inference on the parameters and entropy of IWD under progressive first-failure censoring has been considered. Both the maximum likelikood estimation and Bayesian estimation are investigated. For Bayesian estimation, we apply the Lindley and Importance Sampling method to approximate the Bayesian estimates under both asymmetric and symmetric loss functions. We construct the approximate intervals based on MLEs and Log-transformed MLEs. In addition, we use the Importance Sampling method to derive the HPD intervals. Then, we compare the performance of estimates through EV and MSE. Although we have considered the estimation of entropy under progressive first-failure censoring scheme as much as possible, using a similar method, this censoring scheme can be widely extended to other more efficient and complex censoring schemes. This direction is still very promising and requires more attention and work.

## Figures and Tables

**Table 1:** Maximum likelihood estimates of α, λ, and entropy when α=2, λ=1, H=1.172676.

*k*	*n*	*m*	Scheme	$\hat{\alpha }$		$\hat{\lambda }$		$\hat{H}$	
				EV	MSE	EV	MSE	EV	MSE
1	50	25	(25, 0*24)	2.0608	0.1032	1.0144	0.0452	1.1558	0.0670
			((5, 0*4)*5)	2.1496	0.1788	0.9940	0.0309	1.1029	0.0840
			(0*24, 25)	2.0880	0.0932	1.0017	0.0342	1.1302	0.0535
	50	40	(10, 0*39)	2.0845	0.0734	0.9989	0.0271	1.1271	0.0406
			((2, 0*7)*5)	2.0743	0.0813	0.9888	0.0227	1.1327	0.0467
			(0*39, 10)	2.0470	0.0534	1.0097	0.0189	1.1550	0.0309
	70	35	(35, 0*34)	2.1255	0.0880	1.0047	0.0383	1.1063	0.0537
			((5, 0*4)*7)	2.0550	0.0552	1.0227	0.0210	1.1565	0.0326
			(0*34, 35)	2.0575	0.0758	0.9953	0.0193	1.1458	0.0423
		60	(10, 0*59)	2.0653	0.0517	0.9864	0.0232	1.1307	0.0330
			((2, 0*11)*5)	2.0330	0.0428	1.0140	0.0201	1.1644	0.0281
			(0*59, 10)	2.0573	0.0430	1.0016	0.0179	1.1418	0.0245
2	50	25	(25, 0*24)	2.1001	0.0966	0.9731	0.0251	1.1107	0.0535
			((5, 0*4)*5)	2.1413	0.1461	0.9711	0.0244	1.0936	0.0740
			(0*24, 25)	2.1629	0.2070	0.9762	0.0291	1.0930	0.0972
	50	40	(10, 0*39)	2.0889	0.0698	0.9832	0.0206	1.1182	0.0393
			((2, 0*7)*5)	2.0724	0.0711	0.9806	0.0201	1.1295	0.0453
			(0*39, 10)	2.1180	0.0805	0.9901	0.0150	1.1059	0.0405
	70	35	(35, 0*34)	2.0745	0.0601	0.9968	0.0180	1.1331	0.0359
			((5, 0*4)*7)	2.0583	0.0724	0.9984	0.0214	1.1493	0.0508
			(0*34, 35)	2.0766	0.0677	0.9763	0.0211	1.1251	0.0474
		60	(10, 0*59)	2.0632	0.0433	0.9939	0.0157	1.1364	0.0290
			((2, 0*11)*5)	2.0334	0.0437	0.9965	0.0109	1.1578	0.0274
			(0*59, 10)	2.0729	0.0486	1.0039	0.0108	1.1358	0.0253

**Table 2:** Bayes estimates under squared error loss function of α, λ, and entropy based on the Lindley method when α=2, λ=1, H=1.172676.

*k*	*n*	*m*	Scheme	${\hat{\alpha }}_{S}$		${\hat{\lambda }}_{S}$		${\hat{H}}_{S}$	
				EV	MSE	EV	MSE	EV	MSE
1	50	25	(25, 0*24)	2.2344	0.2017	0.9661	0.0588	1.0599	0.0921
			((5, 0*4)*5)	2.1080	0.1418	0.9837	0.0426	1.1472	0.0803
			(0*24, 25)	2.0959	0.1121	1.0408	0.0314	1.1760	0.0580
	50	40	(10, 0*39)	2.0758	0.0733	1.0173	0.0306	1.1606	0.0408
			((2, 0*7)*5)	2.1015	0.1029	0.9813	0.0245	1.1317	0.0463
			(0*39, 10)	2.0258	0.0545	1.0285	0.0202	1.1950	0.0309
	70	35	(35, 0*34)	2.1470	0.1065	0.9908	0.0343	1.1081	0.0520
			((5, 0*4)*7)	2.0725	0.0526	0.9906	0.0245	1.1497	0.0341
			(0*34, 35)	2.0965	0.0957	1.0092	0.0197	1.1497	0.0434
		60	(10, 0*59)	2.0641	0.0629	1.0127	0.0145	1.1597	0.0296
			((2, 0*11)*5)	2.0570	0.0494	1.0030	0.0213	1.1562	0.0282
			(0*59, 10)	2.0804	0.0583	1.0096	0.0193	1.1446	0.0271
2	50	25	(25, 0*24)	2.2127	0.1435	0.9194	0.0337	1.0417	0.0718
			((5, 0*4)*5)	2.2911	0.2474	0.8959	0.0544	0.9955	0.1181
			(0*24, 25)	2.1768	0.2299	0.9492	0.0426	1.0994	0.1140
	50	40	(10, 0*39)	2.0954	0.0742	0.9941	0.0238	1.1370	0.0416
			((2, 0*7)*5)	2.0976	0.0861	0.9950	0.0208	1.1396	0.0455
			(0*39, 10)	2.0270	0.0710	0.9985	0.0158	1.1846	0.0403
	70	35	(35, 0*34)	2.1225	0.0827	0.9672	0.0296	1.1084	0.0520
			((5, 0*4)*7)	2.2037	0.1643	0.9314	0.0271	1.0547	0.0783
			(0*34, 35)	2.2001	0.1821	0.9282	0.0288	1.0602	0.0865
		60	(10, 0*59)	2.0760	0.0456	0.9823	0.0111	1.1343	0.0247
			((2, 0*11)*5)	2.0626	0.0355	0.9911	0.0115	1.1456	0.0216
			(0*59, 10)	2.0435	0.0336	0.9932	0.0124	1.1584	0.0219

**Table 3:** Bayes estimates under Linex loss function of α, λ, and entropy based on Lindley method when α=2, λ=1, H=1.172676, p=0.5.

*k*	*n*	*m*	Scheme	${\hat{\alpha }}_{L}$		${\hat{\lambda }}_{L}$		${\hat{H}}_{L}$	
				EV	MSE	EV	MSE	EV	MSE
1	50	25	(25, 0*24)	2.2130	0.1582	0.9521	0.0410	1.01956	0.0779
			((5, 0*4)*5)	2.0874	0.1221	1.0328	0.0403	1.1179	0.0812
			(0*24, 25)	2.0444	0.0899	1.0462	0.0178	1.1481	0.0492
	50	40	(10, 0*39)	2.0680	0.0751	1.0147	0.0218	1.1304	0.0401
			((2, 0*7)*5)	2.0646	0.0749	0.9963	0.0287	1.1206	0.0508
			(0*39, 10)	2.0381	0.0577	1.0329	0.0239	1.1528	0.0326
	70	35	(35, 0*34)	2.1109	0.0786	0.9403	0.0384	1.0715	0.0534
			((5, 0*4)*7)	2.0306	0.0765	1.0153	0.0280	1.1483	0.0530
			(0*34, 35)	1.9981	0.0595	1.0039	0.0179	1.1604	0.0438
		60	(10, 0*59)	2.0524	0.0427	1.0046	0.0216	1.1334	0.0251
			((2, 0*11)*5)	2.0499	0.0486	1.0067	0.0167	1.1384	0.0307
			(0*59, 10)	2.0310	0.0338	1.0409	0.0161	1.1648	0.0194
2	50	25	(25, 0*24)	2.2816	0.2478	0.9043	0.0398	0.9843	0.1064
			((5, 0*4)*5)	2.1778	0.1469	0.9258	0.0383	1.0027	0.1004
			(0*24, 25)	2.2197	0.2339	0.9383	0.0437	0.9691	0.1609
	50	40	(10, 0*39)	2.0708	0.0677	0.9765	0.0183	1.1097	0.0423
			((2, 0*7)*5)	2.0240	0.0883	0.9993	0.0232	1.1492	0.0593
			(0*39, 10)	2.0107	0.0686	1.0028	0.0151	1.1563	0.0460
	70	35	(35, 0*34)	2.1340	0.0954	0.9516	0.0280	1.0795	0.0574
			((5, 0*4)*7)	2.1215	0.0939	0.9524	0.0264	1.0562	0.0713
			(0*34, 35)	2.1197	0.1146	0.9626	0.0204	1.0481	0.0754
		60	(10, 0*59)	2.0395	0.0466	1.0138	0.0136	1.1510	0.0308
			((2, 0*11)*5)	2.0500	0.0510	0.9821	0.0150	1.1255	0.0376
			(0*59, 10)	2.0368	0.0402	1.0013	0.0126	1.1407	0.0261

**Table 4:** Bayes estimates under Linex loss function of α, λ, and entropy based on Lindley method when α=2, λ=1, H=1.172676, p=1.

*k*	*n*	*m*	Scheme	${\hat{\alpha }}_{L}$		${\hat{\lambda }}_{L}$		${\hat{H}}_{L}$	
				EV	MSE	EV	MSE	EV	MSE
1	50	25	(25, 0*24)	2.1496	0.1350	0.9629	0.0401	1.0428	0.0820
			((5, 0*4)*5)	2.0804	0.1463	0.9815	0.0299	1.0748	0.0918
			(0*24, 25)	2.0503	0.1180	0.9795	0.0242	1.0841	0.0776
	50	40	(10, 0*39)	2.0666	0.0616	0.9813	0.0219	1.0951	0.0386
			((2, 0*7)*5)	1.9808	0.0456	1.0134	0.0264	1.1622	0.0361
			(0*39, 10)	1.9998	0.0621	0.9873	0.0212	1.1399	0.0387
	70	35	(35, 0*34)	2.0950	0.0773	0.9541	0.0268	1.0763	0.0522
			((5, 0*4)*7)	2.0371	0.0789	1.0013	0.0279	1.1188	0.0582
			(0*34, 35)	2.0219	0.0803	0.9998	0.0227	1.1258	0.0527
		60	(10, 0*59)	2.0313	0.0506	1.0027	0.0198	1.1376	0.0315
			((2, 0*11)*5)	2.0001	0.0318	1.0277	0.0148	1.1677	0.0204
			(0*59, 10)	2.0261	0.0351	0.9998	0.0165	1.1373	0.0246
2	50	25	(25, 0*24)	2.2680	0.2586	0.9074	0.0519	0.9806	0.1179
			((5, 0*4)*5)	2.2524	0.2212	0.8923	0.0428	0.9330	0.1395
			(0*24, 25)	2.0967	0.1157	0.9810	0.0261	1.0361	0.0977
	50	40	(10, 0*39)	2.0814	0.0862	0.9833	0.0188	1.0953	0.0521
			((2, 0*7)*5)	2.0593	0.0611	0.9871	0.0182	1.0984	0.0439
			(0*39, 10)	2.0500	0.0721	0.9917	0.0180	1.1071	0.0463
	70	35	(35, 0*34)	2.1056	0.0793	0.9481	0.0263	1.0777	0.0511
			((5, 0*4)*7)	2.1197	0.0956	0.9439	0.0253	1.0379	0.0719
			(0*34, 35)	2.1511	0.1295	0.9289	0.0283	0.9942	0.1018
		60	(10, 0*59)	2.0718	0.0585	0.9880	0.0112	1.1082	0.0341
			((2, 0*11)*5)	2.0307	0.0325	0.9969	0.0157	1.1305	0.0275
			(0*59, 10)	2.0047	0.0377	0.9980	0.0086	1.1502	0.0244

**Table 5:** Bayes estimates under general entropy loss function of α, λ, and entropy based on Lindley method when α=2, λ=1, H=1.172676, q=−1.

*k*	*n*	*m*	Scheme	${\hat{\alpha }}_{E}$		${\hat{\lambda }}_{E}$		${\hat{H}}_{E}$	
				EV	MSE	EV	MSE	EV	MSE
1	50	25	(25, 0*24)	2.1851	0.1656	0.9729	0.0465	1.0717	0.0836
			((5, 0*4)*5)	2.1052	0.1308	1.0073	0.0338	1.1198	0.0752
			(0*24, 25)	2.0534	0.1109	1.0234	0.0261	1.1560	0.0658
	50	40	(10, 0*39)	2.0594	0.0689	1.0143	0.0261	1.1506	0.0413
			((2, 0*7)*5)	2.0614	0.0712	1.0119	0.0245	1.1462	0.0415
			(0*39, 10)	2.0633	0.0681	1.0261	0.0240	1.1509	0.0370
	70	35	(35, 0*34)	2.1183	0.0919	0.9937	0.0335	1.1141	0.0537
			((5, 0*4)*7)	2.1049	0.0935	0.9901	0.0237	1.1083	0.0551
			(0*34, 35)	2.0446	0.0722	1.0231	0.0193	1.1599	0.0425
		60	(10, 0*59)	2.0523	0.0465	1.0110	0.0181	1.1503	0.0277
			((2, 0*11)*5)	2.0430	0.0464	1.0095	0.0178	1.1542	0.0271
			(0*59, 10)	2.0618	0.0485	1.0082	0.0148	1.1421	0.0259
2	50	25	(25, 0*24)	2.2669	0.2137	0.9171	0.0473	1.0112	0.1030
			((5, 0*4)*5)	2.2491	0.2490	0.9215	0.0479	0.9851	0.1427
			(0*24, 25)	2.1992	0.2334	0.9415	0.0398	1.0039	0.1497
	50	40	(10, 0*39)	2.0818	0.0682	0.9962	0.0212	1.1280	0.0425
			((2, 0*7)*5)	2.0767	0.0692	0.9840	0.0183	1.1189	0.0447
			(0*39, 10)	2.0587	0.0744	1.0054	0.0185	1.1421	0.0470
	70	35	(35, 0*34)	2.1613	0.1117	0.9523	0.0303	1.0780	0.0605
			((5, 0*4)*7)	2.1648	0.1226	0.9517	0.0281	1.0462	0.0780
			(0*34, 35)	2.1292	0.1189	0.9641	0.0220	1.0611	0.0780
		60	(10, 0*59)	2.0542	0.0417	0.9938	0.0124	1.1399	0.0258
			((2, 0*11)*5)	2.0565	0.0438	0.9956	0.0132	1.1369	0.0291
			(0*59, 10)	2.0348	0.0379	1.0018	0.0105	1.1532	0.0247

**Table 6:** Bayes estimates under general entropy loss function of α, λ, and entropy based on Lindley method when α=2, λ=1, H=1.172676, q=1.

*k*	*n*	*m*	Scheme	${\hat{\alpha }}_{E}$		${\hat{\lambda }}_{E}$		${\hat{H}}_{E}$	
				EV	MSE	EV	MSE	EV	MSE
1	50	25	(25, 0*24)	2.1650	0.1665	0.9330	0.0504	1.0151	0.0994
			((5, 0*4)*5)	2.0679	0.1155	0.9738	0.0313	1.0612	0.0821
			(0*24, 25)	2.0317	0.1087	0.9940	0.0260	1.0870	0.0766
	50	40	(10, 0*39)	2.0382	0.0669	0.9939	0.0272	1.1139	0.0441
			((2, 0*7)*5)	2.0330	0.0671	0.9970	0.0249	1.1153	0.0448
			(0*39, 10)	2.0324	0.0637	1.0074	0.0252	1.1214	0.0418
	70	35	(35, 0*34)	2.0978	0.0928	0.9587	0.0344	1.0717	0.0625
			((5, 0*4)*7)	2.0388	0.0741	0.9898	0.0233	1.1017	0.0532
			(0*34, 35)	2.0043	0.0717	1.0062	0.0193	1.1306	0.0500
		60	(10, 0*59)	2.0321	0.0459	0.9930	0.0182	1.1275	0.0297
			((2, 0*11)*5)	2.0211	0.0442	0.9973	0.0166	1.1354	0.0297
			(0*59, 10)	2.0218	0.0409	0.9885	0.0150	1.1301	0.0261
2	50	25	(25, 0*24)	2.2187	0.2059	0.9119	0.0460	1.0028	0.1030
			((5, 0*4)*5)	2.2251	0.2610	0.8957	0.0460	0.9578	0.1374
			(0*24, 25)	2.1907	0.3101	0.9161	0.0445	0.9604	0.1458
	50	40	(10, 0*39)	2.0566	0.0584	0.9736	0.0199	1.0934	0.0436
			((2, 0*7)*5)	2.0458	0.0657	0.9806	0.0185	1.0978	0.0485
			(0*39, 10)	2.0331	0.0610	0.9853	0.0172	1.1046	0.0460
	70	35	(35, 0*34)	2.1365	0.0959	0.9330	0.0282	1.0494	0.0610
			((5, 0*4)*7)	2.1308	0.1119	0.9401	0.0275	1.0275	0.0790
			(0*34, 35)	2.0924	0.1064	0.9493	0.0247	1.0351	0.0814
		60	(10, 0*59)	2.0312	0.0370	0.9843	0.0126	1.1230	0.0276
			((2, 0*11)*5)	2.0426	0.0439	0.9803	0.0132	1.1110	0.0326
			(0*59, 10)	2.0048	0.0361	0.9973	0.0109	1.1427	0.0258

**Table 7:** Bayes estimates of entropy using Importance Sampling when α=2, λ=1, H=1.172676.

*k*	*n*	*m*	Scheme	$\hat{\alpha }$		$\hat{\lambda }$		$\hat{H}$	
				EV	MSE	EV	MSE	EV	MSE
1	50	20	(25, 0*24)	1.9669	0.0354	1.0756	0.0442	1.2513	0.0515
			((5, 0*4)*5)	2.1832	0.1383	0.9757	0.0158	1.0812	0.0541
			(0*24, 25)	2.3923	0.2330	0.7339	0.0888	0.8277	0.1595
		40	(10, 0*39)	2.0855	0.0981	0.9509	0.0273	1.1185	0.0581
			((2, 0*7)*5)	1.9456	0.0653	1.0425	0.0276	1.2487	0.0469
			(0*39, 10)	2.0585	0.0842	0.9507	0.0112	1.1295	0.0404
	70	35	(35, 0*34)	1.9673	0.0651	1.0978	0.0322	1.2603	0.0394
			((5, 0*4)*7)	2.0998	0.0486	0.9782	0.0276	1.1113	0.0344
			(0*34, 35)	2.5157	0.4768	0.5967	0.1736	0.7008	0.2817
		60	(10, 0*59)	2.0269	0.0396	0.9868	0.0126	1.1607	0.0241
			(2, 0*11)*5)	2.0078	0.0532	1.0068	0.0168	1.1859	0.0332
			(0*59, 10)	2.0724	0.0658	1.0103	0.0211	1.1454	0.0411
2	50	20	(25, 0*24)	2.1260	0.2079	0.9434	0.0668	1.1016	0.1009
			((5, 0*4)*5)	2.6489	0.5789	0.6052	0.1803	0.6469	0.3286
			(0*24, 25)	2.9631	1.1193	0.4039	0.3610	0.3852	0.6440
		40	(10, 0*39)	2.1428	0.0810	0.9432	0.0319	1.0652	0.0461
			((2, 0*7)*5)	2.3493	0.1862	0.7244	0.0821	0.8441	0.1309
			(0*39, 10)	2.6610	0.5367	0.5761	0.1890	0.6142	0.3376
	70	35	(35, 0*34)	2.1796	0.1064	0.8989	0.0276	1.0292	0.0588
			((5, 0*4)*7)	2.6541	0.6279	0.5527	0.2119	0.6106	0.3630
			(0*34, 35)	3.4169	2.2878	0.3225	0.4658	0.1931	0.9953
		60	(10, 0*59)	2.3722	0.2157	0.8339	0.0343	0.8916	0.1065
			(2, 0*11)*5)	2.3814	0.2175	0.7520	0.0709	0.8410	0.1383
			(0*59, 10)	2.5075	0.3113	0.5588	0.1969	0.6621	0.2744

**Table 8:** Bayes estimates under squared error loss function of α, λ, and entropy based on Lindley method when α=2, λ=1, *H* = 1.172676, *a* = 0, *b* = 0.

*k*	*n*	*m*	Scheme	${\hat{\alpha }}_{S}$		${\hat{\lambda }}_{S}$		${\hat{H}}_{S}$	
				EV	MSE	EV	MSE	EV	MSE
1	50	25	(25, 0*24)	2.2082	0.1731	0.9569	0.0536	1.0672	0.0787
			((5, 0*4)*5)	2.1024	0.1236	0.9973	0.0416	1.1521	0.0662
			(0*24, 25)	2.0783	0.1237	1.0246	0.0238	1.1815	0.0582
	50	40	(10, 0*39)	2.0949	0.0851	1.023	0.0292	1.1532	0.0451
			((2, 0*7)*5)	2.0902	0.0972	0.9888	0.0285	1.1425	0.0508
			(0*39, 10)	2.0758	0.0824	1.0027	0.0221	1.1562	0.0428
	70	35	(35, 0*34)	2.1123	0.1186	0.9734	0.0373	1.1249	0.0657
			((5, 0*4)*7)	2.0675	0.0990	1.0265	0.0318	1.1784	0.0568
			(0*34, 35)	2.1062	0.0865	0.9807	0.0240	1.1291	0.0463
		60	(10, 0*59)	2.0682	0.0551	1.0043	0.0167	1.1510	0.0276
			((2, 0*11)*5)	2.0541	0.0431	0.9952	0.0156	1.1539	0.0247
			(0*59, 10)	2.0748	0.0538	1.0180	0.0165	1.1509	0.0222
2	50	25	(25, 0*24)	2.2764	0.2065	0.9113	0.0472	1.0064	0.0986
			((5, 0*4)*5)	2.2915	0.2881	0.9119	0.0457	1.0105	0.1247
			(0*24, 25)	2.3071	0.3355	0.9011	0.0517	1.0025	0.1374
	50	40	(10, 0*39)	2.1238	0.0700	0.9925	0.0168	1.1177	0.0346
			((2, 0*7)*5)	2.1110	0.0938	0.9750	0.0207	1.1219	0.0469
			(0*39, 10)	2.0765	0.0510	0.9861	0.0173	1.1425	0.0307
	70	35	(35, 0*34)	2.1629	0.1071	0.9576	0.0288	1.0813	0.0565
			((5, 0*4)*7)	2.1607	0.1463	0.9504	0.0327	1.0879	0.0772
			(0*34, 35)	2.2537	0.2629	0.9120	0.0419	1.0277	0.1145
		60	(10, 0*59)	2.0990	0.0515	0.9743	0.0140	1.1164	0.0283
			((2, 0*11)*5)	2.0752	0.0477	0.9701	0.0127	1.1294	0.0269
			(0*59, 10)	2.0546	0.0430	0.9995	0.0117	1.1562	0.0242

**Table 9:** Bayes estimates of entropy using Importance Sampling procedure when α=2, λ=1, H=1.172676, *a* = 0, *b* = 0.

*k*	*n*	*m*	Scheme	$\hat{\alpha }$		$\hat{\lambda }$		$\hat{H}$	
				EV	MSE	EV	MSE	EV	MSE
1	50	20	(25, 0*24)	2.1546	0.0591	0.9475	0.0543	1.0537	0.0301
			((5, 0*4)*5)	2.1046	0.1199	0.9566	0.0590	1.1089	0.0660
			(0*24, 25)	2.7585	0.8718	0.6468	0.1665	0.6350	0.3742
		40	(10, 0*39)	2.0418	0.0899	1.0567	0.0224	1.1962	0.0442
			((2, 0*7)*5)	1.8975	0.0288	0.9704	0.0148	1.2355	0.0202
			(0*39, 10)	2.1062	0.0727	0.9991	0.0073	1.1174	0.0283
	70	35	(35, 0*34)	1.9718	0.1112	0.9444	0.0184	1.1922	0.0584
			((5, 0*4)*7)	2.3056	0.1741	0.7991	0.0659	0.9122	0.1166
			(0*34, 35)	2.7018	0.7250	0.6630	0.1638	0.6451	0.3327
		60	(10, 0*59)	2.0047	0.0227	0.9513	0.0100	1.1534	0.0145
			(2, 0*11)*5)	2.1170	0.0385	1.0260	0.0253	1.1187	0.0257
			(0*59, 10)	2.2110	0.0863	0.9931	0.0333	1.0456	0.0425
2	50	20	(25, 0*24)	1.9963	0.0733	0.9162	0.0192	1.1582	0.0606
			((5, 0*4)*5)	2.4657	0.5400	0.6098	0.1706	0.7529	0.2983
			(0*24, 25)	3.3960	2.3295	0.3504	0.4319	0.2260	0.9416
		40	(10, 0*39)	2.1716	0.1174	0.8572	0.0299	1.0177	0.0751
			((2, 0*7)*5)	2.3431	0.1821	0.7332	0.0891	0.8470	0.1384
			(0*39, 10)	2.8361	0.8668	0.5793	0.1938	0.5556	0.4241
	70	35	(35, 0*34)	2.1448	0.1080	0.8393	0.0426	1.0286	0.0901
			((5, 0*4)*7)	2.7725	0.9165	0.5504	0.2145	0.5769	0.4258
			(0*34, 35)	2.2137	0.0890	0.7790	0.0574	0.9444	0.0715
		60	(10, 0*59)	2.2492	0.1525	0.8417	0.0456	0.9597	0.0910
			(2, 0*11)*5)	2.2767	0.1454	0.7159	0.0887	0.8728	0.1198
			(0*59, 10)	2.7964	0.6670	0.5519	0.2037	0.5436	0.4008

**Table 10:** Average length and coverage probability of 95% asymptotic intervals/highest posterior density credible intervals of paramater α and entropy when α=2, λ=1, H=1.172676, *k* = 1.

*n*	*m*	Scheme	${\hat{H}}_{ML}$		${\hat{H}}_{ML}\left(Log\right)$		${\hat{H}}_{IS}$	
			AL	CP	AL	CP	AL	CP
50	25	(25, 0*24)	1.0168	0.954	1.0665	0.940	0.9695	0.936
		((5, 0*4)*5)	1.0244	0.972	1.0820	0.958	0.7538	0.824
		(5*5, 0*20)	1.0409	0.950	1.1055	0.934	0.9725	0.948
50	40	(10, 0*39)	0.8054	0.950	0.8264	0.952	0.7751	0.936
		((2, 0*7)*5)	0.7991	0.962	0.8198	0.942	0.7532	0.936
		(2*5, 0*35)	0.8086	0.956	0.8306	0.948	0.7769	0.952
70	35	(35, 0*34)	0.8523	0.946	0.8778	0.942	0.8176	0.938
		((7, 0*6)*5)	0.8666	0.964	0.8933	0.962	0.8216	0.952
		(5*7, 0*28)	1.0202	0.952	0.8694	0.954	0.8202	0.936
70	60	(10, 0*59)	0.6554	0.962	0.6657	0.960	0.6344	0.944
		((2, 0*11)*5)	0.6515	0.946	0.6617	0.950	0.6230	0.936
		(2*5, 0*55)	0.6541	0.960	0.6642	0.958	0.6342	0.944

**Table 11:** Summary for model fit using −lnL, K-S statistic and associated *p*-value.

Distribution	MLEs	lnL	K-S	*p*-Value
Inverse Weibull	$\left(\hat{\alpha },\hat{\lambda }\right)=\left(1.415,283.837\right)$	395.649	0.152	0.0728836

**Table 12:** Progressive first-failure censored sample in the given censoring scheme when k=2, n=36, m=18.

Scheme	Sample
R1 = (18, 0*17)	12, 24, 32, 32, 34, 38, 54, 55, 58, 60, 61, 65, 68, 70, 91, 109, 110, 143
R2 = (1*18)	12, 15, 22, 24, 32, 32, 33, 34, 38, 43, 44, 54, 55, 58, 60, 65, 68, 70
R3 = (0*17, 18)	12, 15, 22, 24, 32, 32, 33, 34, 38, 38, 43, 44, 48, 52, 54, 55, 56, 58

**Table 13:** Point and interval estimation of parameters and entropy using MLE and Bayes methods.

Estimates	R1	R2	R3
${\hat{\alpha }}_{ML}$	1.17 (0.87, 1.59)	1.073 (0.78, 1.48)	0.95 (0.68, 1.33)
${\hat{\lambda }}_{ML}$	123.79 (33.88, 452.35)	88.46 (26.28, 297.76)	61.08 (19.07, 195.67)
${\hat{H}}_{ML}$	6.01 (5.36, 6.75)	6.22 (5.50, 7.04)	6.57 (5.77, 7.48)
${\hat{\alpha }}_{IS}$	1.10 (0.94, 1.42)	1.13 (0.98, 1.38)	1.20 (1.14, 1.21)
${\hat{\lambda }}_{IS}$	123.47 (42.40, 304.71)	100.64 (45.80, 205.23)	99.12 (72.85, 106.27)
${\hat{H}}_{IS}$	6.30 (5.56, 7.18)	5.68 (5.68, 5.68)	5.52 (5.36, 5.55)
